# Analysis of Risk Factors for 24 Patients With COVID-19 Developing From Moderate to Severe Condition

**DOI:** 10.3389/fcimb.2020.548582

**Published:** 2020-09-15

**Authors:** Dianming Li, Chuanmiao Liu, Jiahui Liu, Junfeng Hu, Yanli Yang, Yufu Zhou

**Affiliations:** ^1^Department of Respiratory and Critical Care Medicine, The First Affiliated Hospital of Bengbu Medical College, Bengbu, China; ^2^Department of Infectious Diseases, The First Affiliated Hospital of Bengbu Medical College, Bengbu, China; ^3^Department of Hematology, The First Affiliated Hospital of Bengbu Medical College, Bengbu, China; ^4^Department of Tumor Radiotherapy, The First Affiliated Hospital of Bengbu Medical College, Bengbu, China

**Keywords:** COVID-19, moderate, severe, risk factors, IL-6

## Abstract

**Objective:** The present study aimed at investigating the clinical risk factors for COVID-19 patients developing from moderate condition to severe condition, and providing reference for early intervention and prognosis.

**Methods:** We collected the clinical data of 24 patients with moderate-to-severe COVID-19 who were admitted to the isolation ward of the First Affiliated Hospital of Bengbu Medical College from January, 2020 to February 20, 2020, and evaluated the data of clinical characteristics, blood test results, inflammatory index, chest CT imaging characteristics, and antiviral treatment, comparing this with the clinical data of 41 patients with moderate condition in the same period. From this comparison we thus summarized the current knowledge of potential risk factors for COVID-19 patients developing from moderate to severe condition.

**Results:** (1) Clinical characteristics: The moderate-to-severe group and the moderate group in terms of combined common underlying diseases and respiratory frequency showed significant difference statistically (*t*-value were 13.32, 6.17, respectively, *P* < 0.05), while no significant difference between the two groups in gender, age, or clinical symptoms was statistically observed(*P* > 0.05).

(2) Analysis of blood test results: The lymphocyte count and plasma albumin of the moderate-to-severe group were significantly lower than those of the moderate group (*t*-values were 4.16, 4.11, respectively, *P* < 0.05), and the blood glucose and urea of the moderate-to-severe group were significantly higher than those of the moderate group (*t*-value were 3.27, 4.19, respectively, *P* < 0.05). However, there was no significant difference in terms of white blood cell count (WBC), platelet count (PLT), and glutamic-pyruvic transaminase (GPT) (*P* > 0.05).

(3) Comparison of inflammatory indicators: The level of IL-6 and CRP of the moderate-to-severe group were significantly higher than those of the moderate group (*t*-values were 2.84, 4.88, respectively, *P* < 0.05).

(4) Imaging comparison: As for patients with moderate COVID-19, the imaging manifestations were the concurrence of ground-glass opacity, patchy shadow, and consolidation shadow in both lungs, diffuse ground-glass opacity in both lungs accompanied by air bronchogram, and large area consolidation of both lungs with pulmonary interstitial changes. The possibility for these patients to develop into severe condition increased, and the differences were statistically significant (*t* = 10.92, *P* < 0.05).

(5) Clinical antiviral treatment: There was no statistically significant difference in the combination of two or three antiviral drugs between the two groups (χ^2^ = 0.05, *P* > 0.05).

**Conclusion:** Current evidence suggested that the combination of common underlying diseases, respiratory frequency, lymphocyte count, blood glucose, albumin, urea level, inflammatory factors (CRP, IL-6), and imaging manifestations collectively contributed to the potential risk factors for the development of COVID-19 from moderate condition to severe condition. Particular attention should be paid to early detection and intervention during clinical work, which will be of vital significance to the ascent of the recovery rate as well as the reduction of mortality.

## Introduction

Since December 2019, patients with novel coronavirus pneumonia have been detected in the city of Wuhan in Hubei province. With the rapid spread of the epidemic, additional cases have been found in other parts of China and abroad. The disease was officially named “2019 coronavirus disease” (COVID-19) by the Director-General of WHO, Tan Desai, on February 11th and a subsequent announcement by the National Center for Disease Control and Prevention declared the inclusion of COVID-19 in the national “class B” infectious diseases and the adoption of “class A” infectious disease prevention and control measures Novel coronavirus pneumonia diagnosis and treatment plan of People's Republic of China national health and Health Committee. Fundamental clinical and epidemiological studies on COVID-19 have been reported recently (Chan J. et al., 2020; Chen L. et al., 2020; Huang et al., 2020; Ren et al., 2020; Wang et al., 2020). With the efforts of positive and effective prevention and control measures throughout the country and the devotion from the vast majority of medical workers, the epidemic has been basically controlled. However, the number of cases has continued to grow dramatically among overseas countries, especially Italy, Spain, and others, accompanied by a relative high mortality rate. We found that some moderate patients tend to develop into severe condition in a short period of time, or even become critical, so it is very difficult to improve the level of treatment. The distinction for these patients in the early stage will be of great value to the improvement of diagnosis and treatment. In the study, we collected clinical data derived from 24 patients admitted to our hospital with COVID-19 developing from moderate to severe condition, compared this with the clinical data of 41 patients with moderate condition in the same period, and analyzed the potential risk factors for COVID-19 patients developing from moderate to severe condition.

## Patients and Methods

### Patients

The study has been approved by the medical ethics committee of the First Affiliated Hospital of Bengbu Medical College, which conforms to the principles of the Declaration of Helsinki. Our hospital is the designated hospital for COVID-19 in Anhui Province, and one of the four intensive treatment bases for severe patients in the province. All the cases meet the COVID-19 diagnostic criteria (sixth edition): (1) epidemiological history: travel history or residential history in Wuhan and its surrounding areas or other communities with reported cases within 14 days before onset; history of contact with a novel coronavirus infected person (positive in nucleic acid test) within 14 days before onset; exposure to patients with fever or respiratory symptoms from Wuhan and its surrounding areas or other communities with reported cases within 14 days before onset; and a clustering onset of disease. (2) Clinical manifestations: fever and/or respiratory symptoms; the aforementioned imaging characteristics of COVID-19; and the total WBC is normal or decreased in the early stage, or the lymphocyte count is decreased. A suspected case can be diagnosed if the patient has any one of the characteristics of epidemiological histories and conforms to any two of the clinical manifestations. If the patient has no epidemiological history and conforms to three of the clinical manifestations, it can be diagnosed as a suspected case. The inclusion criteria for confirmed cases was: suspected cases with one of the following pieces of etiological evidence: positive nucleic acid of novel coronavirus was detected in Real-time fluorescence RT-PCR; or gene sequencing of the virus revealed a high homology with the novel coronavirus. We collected the clinical data of 24 patients with moderate-to-severe COVID-19 who were admitted to the isolation ward of the First Affiliated Hospital of Bengbu Medical College from January, 2020 to February 20, 2020, and evaluated the clinical characteristics, blood test results, inflammatory index, chest CT imaging characteristics, and antiviral treatment, comparing these with the clinical data of 41 patients with moderate condition in the same period, and summarized the potential risk factors for COVID-19 patients developing from moderate to severe condition.

### Clinical Typing of Disease Severity

All confirmed patients were clinically classified according to the “diagnosis and treatment plan of novel coronavirus pneumonia” at admission. Moderate condition was classified as a patient with fever and respiratory symptoms with whom manifestations of pneumonia can be found in imaging findings. Severe condition was classified based on any of the following: respiratory distress, RR ≥ 30 times/min; under resting state, oxygen saturation ≤ 93%; and arterial partial oxygen pressure (PaO_2_)/oxygen concentration (FiO_2_) ≤ 300 mmHg (1 mmHg = 0.133 kpa). Patients with lesion progression more than 50% shown by pulmonary imaging within 24–48 h were managed under severe care Novel coronavirus pneumonia diagnosis and treatment plan of People's Republic of China national health and Health Committee.

### Test Method

In the morning of the second day after admission, 2 ml of the fasting venous blood was taken and sent to the laboratory to check the blood routine and blood biochemistry; automatic blood cell analyzer and blood biochemical analyzer were used for detection. The serum inflammatory factor IL-6 was detected by enzyme-linked immunosorbent assay (ELISA). The operation was carried out in strict accordance with the manual. The kit was purchased from eBioscience company (EPX650-16500-901).

### Statistical Analysis

SPSS 19.0 was used. The counting data of normally distributed measurements was expressed by $\bar{x}$± *s, t*-test was conducted, and the measurement data in percentage were tested by χ^2^/*t*. *P* < 0.05 was considered statistically significant.

## Results

### Comparison of Clinical Characteristics

The general information of the 24 patients developing from moderate to severe condition on admission included: the median age, which was 56 years (21–83 years); 15 males (62.50%) and nine females (37.50%); and 13 patients (54.17%) with one or no common underlying diseases, and 11 patients (45.83%) with more than two kinds of common underlying diseases, such as hypertension, coronary heart disease, diabetes, etc. The 41 moderate patients were 30–78 years old, and the median age was 53 years old; there were 20 male patients (48.78%), and 21 female patients (51.22%); 36 patients (87.80%) had one or no common underlying diseases, and five patients (12.20%) had more than two kinds of common underlying diseases. Among the 24 confirmed patients, symptoms and signs included: 21 patients (87.50%) with fever, eight patients (33.33%) with cough and sputum, three patients (12.50%) with chest tightness and dyspnea, and no patients (0%) with other symptoms. The main physical signs included increased respiratory frequency (≥24 times /min) in eight patients (33.33%). Among the 41 confirmed cases, 41 patients (100%) had fever, 12 patients (29.27%) had cough and expectoration, one patient (2.44%) had chest distress and dyspnea, and one patient (2.44%) had other signs. The main signs included increased respiratory rate (≥24 times/min) in five patients (12.20%). By comparison, there were significant differences in combined common underlying diseases and respiratory frequency between the moderate group and the severe group (*t*-values were 13.32, 6.17, respectively, *P* < 0.05), while there were no significant differences in gender, age, or clinical symptoms between the two groups (*P* > 0.05) (Table 1).

**Table 1:** Comparison of clinical characteristics between two groups.

**General**		**Moderate-**	**Moderate**	***χ^2^*/*t***	***P*-value**
**information**		**to-severe**	**Group**		
		**Group**	**(*N* = 41)**		
		**(*N* = 24)**			
Gender	Male	15	20	1.15	>0.05
	Female	9	21		
Age	<40 yr	1	5	2.52	>0.05
	40–50 yr	4	11		
	51–60 yr	12	15		
	≥60 yr	7	10		
Combined underlying diseases	1	13	36	13.32	<0.05
	≥2	11	5		
Clinical symptoms	Fever	21	41	3.41	>0.05
	Cough/sputum	8	12		
	chest distress and dyspnea	3	1		
	others	0	1		
Main physical signs	<24 times /min	16	36	6.13	<0.05
	≥24 times /min	8	5		

### Comparison of Blood Test Results

The comparison of hematological indexes on the second day after admission of the two groups of patients showed that the lymphocyte count and plasma albumin in the moderate-to-severe group were significantly lower than that in the moderate group (*t*-values were 4.16, 4.11, respectively, *P* < 0.05). The levels of blood glucose and urea in the moderate-to-severe group were significantly higher than the moderate group (*t*-values were 3.27, 4.19, respectively, *P* < 0.05). However, there was no significant difference in terms of white blood cell count (WBC), platelet count (PLT), or glutamic-pyruvic transaminase (GPT) (*P* > 0.05) (Table 2).

**Table 2:** Comparison of hematological indexes between the two groups.

**Hematological**	**Moderate-to-severe**	**Moderate Group**	***t***	***P-*value**
**index**	**Group (*N* = 24)**	**(*N* = 41)**		
WBC (×10^9^/L)	6.34 ± 3.68	6.42 ± 3.53	0.09	>0.05
Lymphocyte count (×10^9^/L)	0.98 ± 0.53	1.56 ± 0.55	4.16	<0.05
PLT (×10^9^/L)	237.58 ± 125.53	257.68 ± 84.12	0.77	>0.05
Plasma albumin (g/L)	35.03 ± 5.92	40.01 ± 3.85	4.11	<0.05
ALT (U/L)	42.88 ± 41.83	36.73 ± 34.71	0.63	>0.05
Blood glucose (mmol/L)	8.84 ± 3.88	6.51 ± 1.86	3.27	<0.05
Urea (umol/L)	5.97 ± 2.96	3.74 ± 1.31	4.19	<0.05

### Comparison of Inflammatory Indicators

The levels of IL-6 and CRP of the moderate-to-severe group were significantly higher than those of the moderate group (*t*-values were 2.84, 4.88, respectively, *P* < 0.05) (Table 3).

**Table 3:** Comparison of inflammatory indicators between the two groups.

**Inflammatory**	**Moderate-to-severe**	**Moderate Group**	***t***	***P*-value**
**indicators**	**Group (*N* = 24)**	**(*N* = 41)**		
IL-6 (pg/ml)	9.88 ± 4.59	6.69 ± 4.23	2.84	<0.01
CRP (mg/L)	47.88 ± 16.63	28.35 ± 14.91	4.88	<0.01

### Comparison of Imaging Manifestation

**Figure 1:**
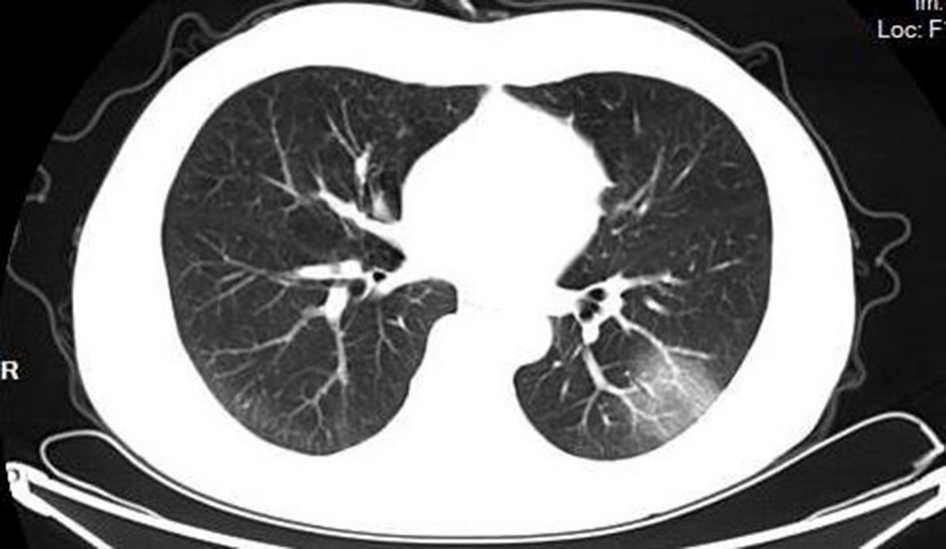
Single ground-glass opacity.

**Figure 2:**
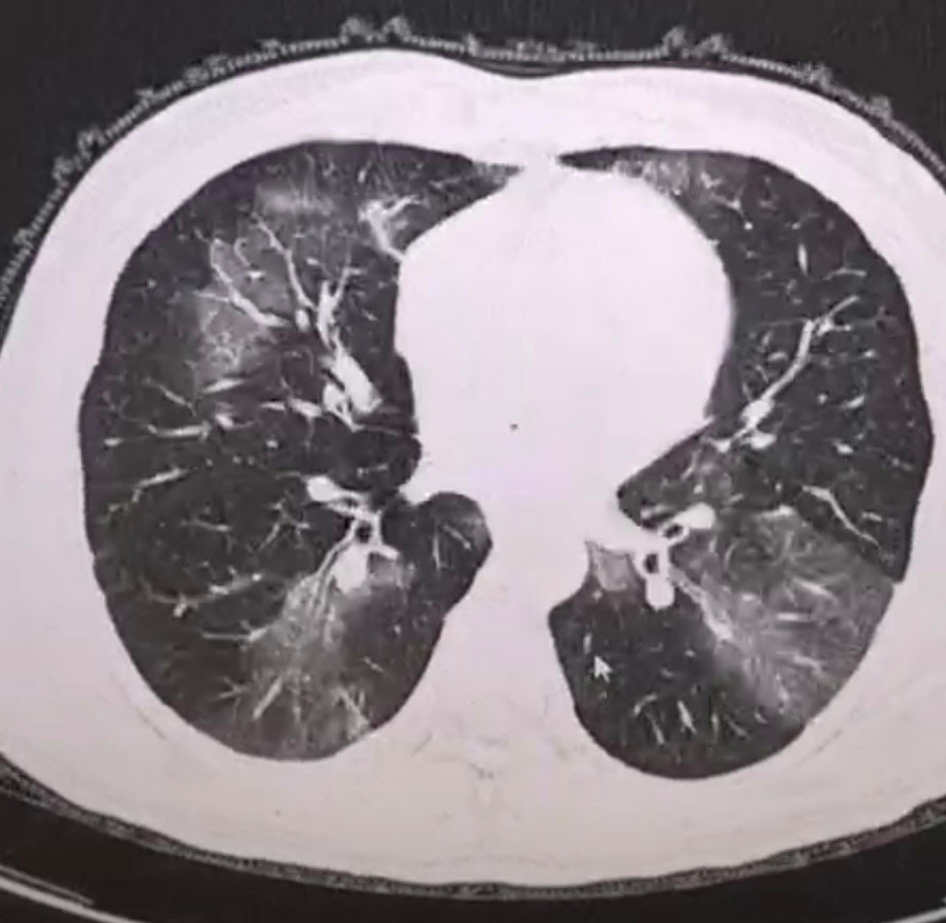
Multiple external subpleural ground-glass opacity.

**Figure 3:**
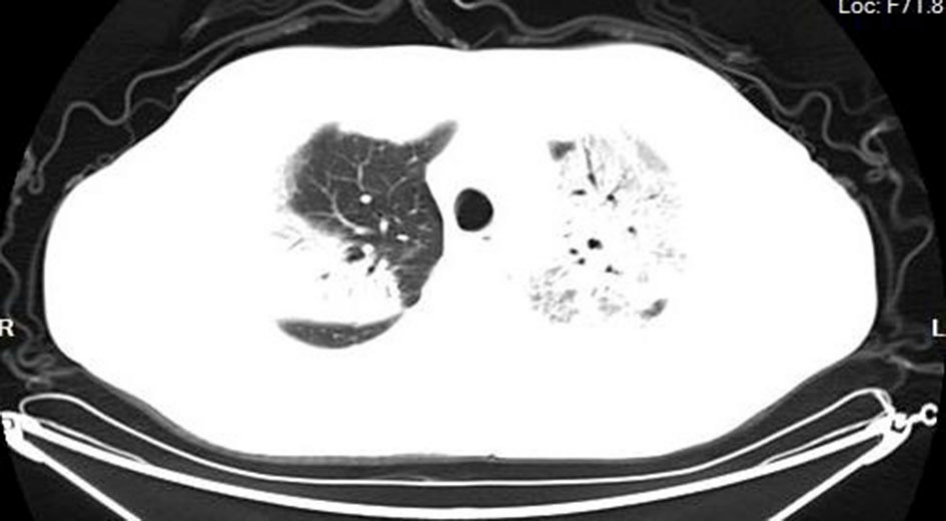
Ground-glass opacity, patchy shadow, and consolidation shadow in both lungs.

**Figure 4:**
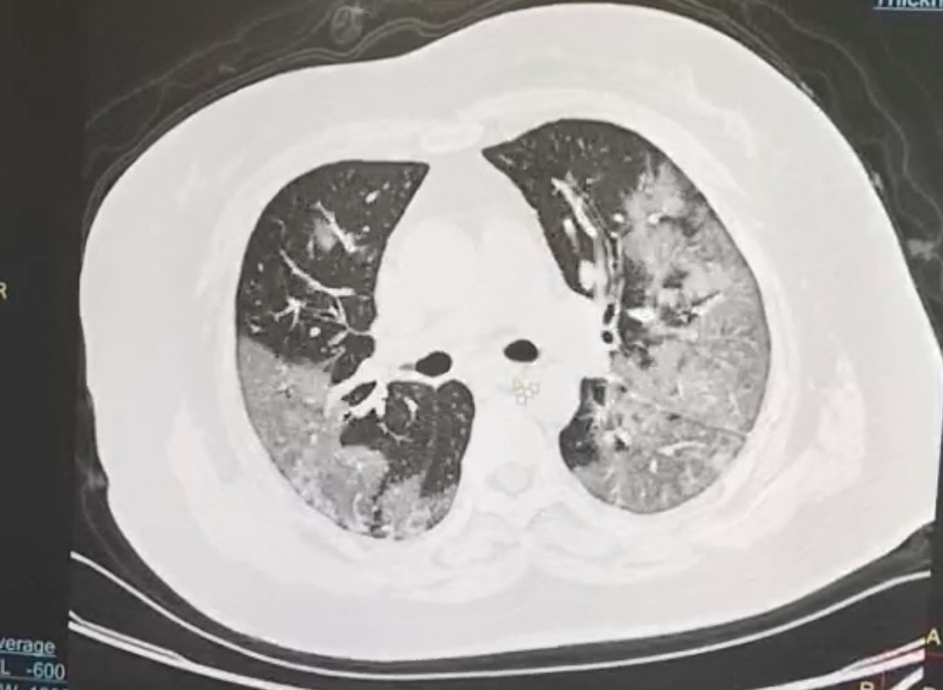
Diffuse ground-glass opacity in both lungs accompanied by air bronchogram.

**Figure 5:**
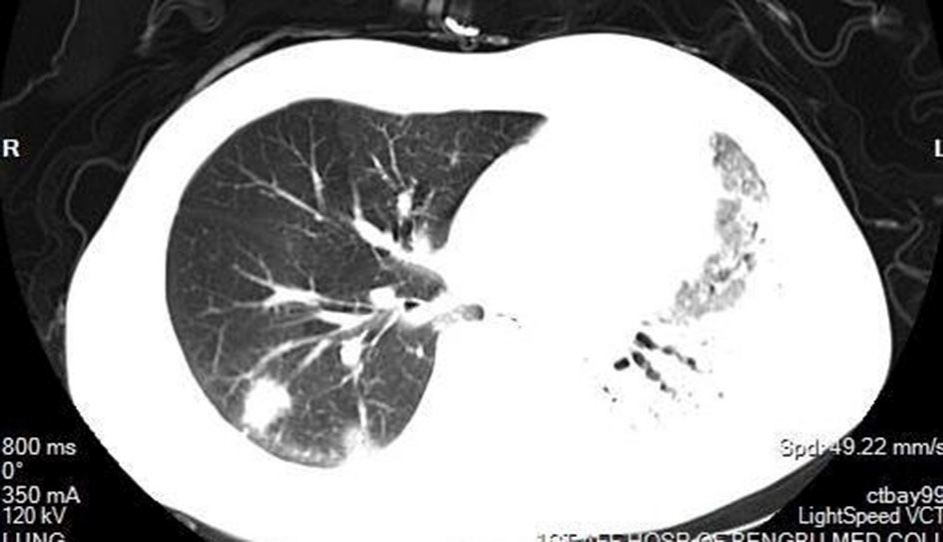
Large area consolidation of both lungs with pulmonary interstitial changes.

According to the CT imaging manifestation of 65 COVID-19 patients, the typical imaging signs were (Figures 1–5): (1) single ground-glass opacity (GGO); (2) multiple external subpleural ground-glass opacity; (3) concurrence of ground-glass opacity, patchy shadow, and consolidation shadow in both lungs; (4) diffuse ground-glass opacity in both lungs accompanied by air bronchogram; and (5) large area consolidation of both lungs with pulmonary interstitial changes. Comparisons of the imaging manifestation between the two groups showed a statistically significant difference (*t* = 10.92, *P* < 0.05). In terms of the proportion of the latter three typical signs, the moderate-to-severe group was higher than the moderate group (Table 4).

**Table 4:** Comparison of imaging manifestation between the two groups.

**Imaging**	**Moderate-to-**	**Moderate**	***t***	***P*-value**
**manifestation**	**severe Group (%)**	**Group (%)**		
Single ground-glass opacity	1 (4.17%)	8 (19.51%)	10.92	<0.05
Multiple external subpleural Ground-glass opacity	4 (16.67%)	17 (41.46%)		
Ground-glass opacity, patchy shadow and consolidation shadow in both lungs	11 (45.83%)	10 (24.39%)		
Diffuse ground-glass opacity in both lungs accompanied by air bronchogram	7 (29.16%)	6 (14.64%)		
Large area consolidation of both lungs with pulmonary interstitial changes	1 (4.17%)	0		

### Comparison of Antiviral Treatment

For clinical antiviral treatment of COVID-19, according to the current scheme, it is recommended to use abidol, klidge, interferon, chloroquine, ribavirin, etc. Two or three used in conjunction are recommended. The dosage, method, and course of treatment are in accordance with the national covid-19 treatment program. This study shows no statistically significant difference in the combination of two antiviral drugs or the combination of three antiviral drugs between the two groups (χ^2^ = 0.05, *P* > 0.05) (Table 5).

**Table 5:** Comparison of antiviral treatment between the two groups.

**Antiviral**	**Moderate-to-**	**Moderate**	***χ^2^***	***P*-value**
**treatment**	**severe Group**	**Group**		
Combination of 2 antiviral treatment	20	35	0.05	>0.05
Combination of 3 antiviral treatment	4	6		

## Discussion

It can be suggested that some of the moderate patients can develop to the severe type, or even to the critical type, within a very short period of time from the clinical work, which makes the improvement of the remedy rate of clinical treatment more difficult. The detection of these patients from the early stage will be worthwhile to the improvement of diagnosis and treatment. This study analyzed the clinical data of 24 COVID-19 patients admitted to our hospital developing from moderate to severe condition, and compared this with the clinical data of 41 moderate patients in the same period; the results were consistent with the results of Guo et al. (2019). The most common underlying diseases are hypertension, diabetes, and coronary heart disease. This may be because the majority of patients are the elderly with a compromised immunity, so we should pay extra attention to elderly patients. However, further analysis of gender, age, and clinical symptoms revealed no significant correlation with the development from moderate to severe condition (*P* > 0.05), which might be attributed to the small number of cases. (2) Compared with the hematological indexes of the two groups after admission, the lymphocyte count and plasma albumin levels in the moderate-to-severe group were significantly lower than those in the moderate group (*t*-values were 4.16, 4.11, respectively, *P* < 0.05). The decrease of lymphocyte count indicates the possibility of immune impairment, and the decrease of albumin may contribute to the hepatic albumin synthesis disorder caused by the direct damage of the virus to hepatocytes. Some researchers (Guo et al., 2019) proposed to use lymphocyte count <0.8 × 10^9^/L as one of the indicators to predict the death risk model of viral pneumonia. The moderate-to-severe group was significantly higher than the moderate group in blood glucose and urea (*t*-values were 3.27, 4.19, respectively, *P* < 0.05), these patients with elevated blood glucose and urea are more likely to develop to severe type. The increase of blood glucose may be connected with the severity of patients or the existence of complicated diabetes, while the increase of BUN may be related to the severe inflammatory response and the hypermetabolism caused by fever in severe patients. However, there was no significant difference in contrast with WBC, PLT, and GBT (*P* > 0.05). It is inferred that moderate patients with a low lymphocyte count and low albumin on admission are more likely to develop to severe condition, which is consistent with the results of most clinical studies (Chen Y. et al., 2020), thus more efforts should be given to these cases. With great individual diversities, we also found that patients had different immune responses to the virus, leading to vast varieties in clinical symptoms, disease progression, and response to therapeutic drugs (Castrucci, 2018). Therefore, for the clinical progression of COVID-19, we should also consider the differences in individual inflammatory responses and search for some objective indicators to help accurately predict the clinical progression and cover the deficiency.

It is well-known that the immune function can enable the body to acquire the defense ability required to resist external infection and eliminate foreign microorganisms, thereby inhibiting the infection and restoring health. But everything has two sides. When the virus invades the body, if the immune system is overactivated or out of control, it will produce an extreme immune response, and release large amounts of cytokines, which in turn attack the host. This phenomenon is called “inflammatory storm.” Numerous evidences have shown that cytokines and chemokines are significantly elevated in patients with severe infection and are considered to reflect the severity of the disease (La Gruta et al., 2007). Studies have shown that the expression levels of serum IL-2R, IL-6, and other cytokines in patients with COVID-19 dramatically increased on average, particularly in the critical patients. Therefore, some scholars pointed out that peripheral blood IL-6 could be applied as a key factor to independently predict the progression of COVID-19. It can be speculated on the basis of that, as a blocking target, IL-6 might have potential clinical value in inhibiting the inflammatory response. Tocilizumab, namely human anti-human interleukin-6 receptor monoclonal antibody, suppresses the activity of IL-6 in peripheral blood to block or reduce the inflammatory response (Yang et al., 2020). With the detection of serum CRP and IL-6 in patients, we found that the levels of IL-6 and CRP in the moderate-to-severe group were significantly higher than that in the moderate group (*t*-values were 2.84, 4.88, respectively, *P* < 0.05), indicating the possibility of immunocompromise. The moderate patients with elevated CRP and IL-6 are more likely to develop into severe condition.

In order to understand the status of lung lesions in COVID-19 patients, we recommend chest HRCT to avoid misdiagnosis and missed diagnosis. Clinical studies have demonstrated that COVID-19 has characteristic manifestations in chest CT. According to the CT imaging manifestations of 65 COVID-19 patients, the comparison of the imaging manifestations between the two groups of patients displayed that the latter three manifestations in the moderate-to-severe group were significantly higher than those in the moderate group (*t* = 10.92, *P* < 0.05). It is necessary to note that COVID-19 patients rarely present with pleural effusion or lymphadenopathy (Medical Expert Group of Tongji Hospital Affiliated to Tongji Medical College of Huazhong University of Science Technology, 2020).

For antiviral treatment, the treatment scheme in “the Diagnosis and Treatment Scheme of the Pneumonia Caused by the Novel Coronavirus” established by the National Health Commission of the People's Republic of China has been constantly adjusted. α- interferon, lopinavir/ritonavir, abidor tablets, ribavirin, and chloroquine phosphate, can be trialed. With the ever-changing treatment courses, the joint application of three or more antiviral drugs is not recommended. For the vast majority of the studied cases, we adopted two antiviral drugs. For a small number of young patients, we tried three antiviral drugs with the consent of patients, which had no apparent effect on preventing the moderate patients from developing into severe condition, and the difference between the two groups was not statistically significant (*P* > 0.05). Meanwhile, the time for the virus to turn negative was not shortened, and a proportion of patients even had adverse reactions. So, we do not recommend the use of more than two antiviral drugs, the exact efficacy of which needs further clinical observation.

In our study, the majority of the 24 moderate-to-severe patients developed into moderate condition after active treatment, one patient developed into critical condition, and all these patients were cured and discharged eventually. In conclusion, the risk factors for COVID-19 patients to develop from moderate to severe condition consists of: complicated common underlying diseases, respiratory frequency, lymphocyte count, blood glucose, albumin, urea, inflammatory factor (CRP, IL-6), and imaging manifestations. It is worth noting in clinical work that early detection and treatment is crucial to raise the cure rate and reduce the mortality. However, due to limited number of cases in this study, it may be necessary to conduct a meta-analysis of the relevant parts in further research to make the results more convincing.

## Data Availability Statement

The datasets presented in this study can be found in online repositories. The names of the repository/repositories and accession number(s) can be found in the article/supplementary material.

## Ethics Statement

The studies involving human participants were reviewed and approved by the First Affiliated Hospital of Bengbu Medical College (2020ky010).

## Author Contributions

DL, YY, CL, and YZ conceived and designed the study. DL, JH, and YZ contributed to the literature search. DL, JL, and YZ contributed to data collection. DL, JH, and YY contributed to data analysis. DL and YY contributed to data interpretation. DL, CL, and JH contributed to the figures. YY and DL contributed to writing of the report. All authors contributed to the article and approved the submitted version.

## Conflict of Interest

The authors declare that the research was conducted in the absence of any commercial or financial relationships that could be construed as a potential conflict of interest.
